# Blood glucose forecasting from temporal and static information in children with T1D

**DOI:** 10.3389/fped.2023.1296904

**Published:** 2023-12-14

**Authors:** Alexander Marx, Francesco Di Stefano, Heike Leutheuser, Kieran Chin-Cheong, Marc Pfister, Marie-Anne Burckhardt, Sara Bachmann, Julia E. Vogt

**Affiliations:** ^1^ Department of Computer Science, ETH Zurich, Zurich, Switzerland; ^2^ Pediatric Pharmacology and Pharmacometrics, University Children’s Hospital Basel, Basel, Switzerland; ^3^ Department of Clinical Research, University Hospital Basel, Basel, Switzerland; ^4^ Pediatric Endocrinolgy and Diabetology, University Children’s Hospital Basel, Basel, Switzerland

**Keywords:** T1D, diabetes, pediatrics, physical activity, forecasting, machine learning, deep learning

## Abstract

**Background:**

The overarching goal of blood glucose forecasting is to assist individuals with type 1 diabetes (T1D) in avoiding hyper- or hypoglycemic conditions. While deep learning approaches have shown promising results for blood glucose forecasting in adults with T1D, it is not known if these results generalize to children. Possible reasons are physical activity (PA), which is often unplanned in children, as well as age and development of a child, which both have an effect on the blood glucose level.

**Materials and Methods:**

In this study, we collected time series measurements of glucose levels, carbohydrate intake, insulin-dosing and physical activity from children with T1D for one week in an ethics approved prospective observational study, which included daily physical activities. We investigate the performance of state-of-the-art deep learning methods for adult data—(dilated) recurrent neural networks and a transformer—on our dataset for short-term (30 min) and long-term (2 h) prediction. We propose to integrate static patient characteristics, such as age, gender, BMI, and percentage of basal insulin, to account for the heterogeneity of our study group.

**Results:**

Integrating static patient characteristics (SPC) proves beneficial, especially for short-term prediction. LSTMs and GRUs with SPC perform best for a prediction horizon of 30 min (RMSE of 1.66 mmol/l), a vanilla RNN with SPC performs best across different prediction horizons, while the performance significantly decays for long-term prediction. For prediction during the night, the best method improves to an RMSE of 1.50 mmol/l. Overall, the results for our baselines and RNN models indicate that blood glucose forecasting for children conducting regular physical activity is more challenging than for previously studied adult data.

**Conclusion:**

We find that integrating static data improves the performance of deep-learning architectures for blood glucose forecasting of children with T1D and achieves promising results for short-term prediction. Despite these improvements, additional clinical studies are warranted to extend forecasting to longer-term prediction horizons.

## 1. Introduction

Type 1 diabetes (T1D) is an endocrine disorder characterized by absolute insulin deficiency caused by the destruction of pancreatic β-cells through autoimmune processes, leading to elevated blood glucose levels (hyperglycemia) if left untreated. Treatment involves administering exogenous insulin to cover both basal insulin requirements and dietary carbohydrates, with individualized adjustments over time. Basal insulin can be delivered through continuous subcutaneous infusion or injections with long-acting insulin. Bolus injections of rapid-acting insulin are used to cover meals and correct high glucose levels. In insulin-treated persons with T1D, low levels of blood glucose (hypoglycemia) is a dreaded acute complication, which in mild forms leads to symptoms such as fatigue, trembling or hunger, whereas episodes of nocturnal hypoglycemia are mostly unrecognized and therefore may persist for hours (1). Severe episodes may manifest as unconsciousness or seizures and may, in rare cases, lead to sudden cardiac death (2, 3).

To reduce hyper- or hypoglycemic excursions, reliable prediction of future blood glucose levels from previous measurements is desirable for children, as well as adults with T1D. Since the release of the OhioT1DM dataset (4), which consists of data of 6, and later 12 (5) adults with T1D, the topic of blood glucose forecasting has been picked up by the machine learning community (6–10). For example, McShinsky and Marshall (7) investigated the performance of classical non deep-learning based methods such as autoregressive moving average (ARIMA), random forests, and support vector machines (SVM) for forecasting blood glucose values. More recently, the community focused on deep-learning architectures such as convolutional neural networks (CNN) with recurrent units (11–13), generative adversarial networks (GANs) (14), deep ensemble methods (15, 16), stacked LSTMs (17, 18), extended GRU networks (19) or methods employing dilation, e.g. dilated CNNs (20) or dilated recurrent neural networks (RNNs) (21) show promising results. Most studies so far focused on adult data (cf. (22–25) for a detailed overview), and it is therefore unclear if these results generalize to children. Although some research has been conducted for children with T1D (26, 27), these works focus on explainable methods for classification and do not study deep-learning approaches. Further, the datasets from children with T1D (28, 29) used in these studies have not been obtained in a controlled/supervised setting with a focus on physical activity and only used CGM data.

This study used data from children who were exercising in a supervised sports camp setting and integrated not only glucose levels but also carbohydrate intake, insulin-dosing and records of physical activity, as well as various patient characteristics. We expect that, compared to data from adults, our data poses two additional challenges: First, the data is more heterogeneous since insulin requirements vary with age and developmental stage of a child (30, 31). Second, the children engaged in different types of physical activity, which has both a direct effect on the blood glucose level due to the energy requirement during the activity (32, 33), as well as a long-lasting effect on the insulin sensitivity (34).

The purpose of our analysis is to investigate whether the success of deep-learning algorithms on adult data transfers to children, where we evaluate RNN architectures (vanilla RNN, GRU, and LSTM), dilated RNNs (which are designed to capture long-ranging dependencies), and a transformer model. Further, we investigate if the integration of static participant information (gender, age, BMI, time since T1D was diagnosed, etc.) can help to calibrate the forecasting algorithms, e.g., to account for age- and weight dependent insulin requirements (31). Lastly, as nocturnal hypoglycemia is most feared and more challenging to prevent, we compare the predictive performance of the considered methods for a complete day to predictions only during the night.

## 2. Materials and methods

### 2.1. Study data

In the following, we describe the study design and population, experimental protocol, and the preprocessing necessary for the machine learning analysis.

#### 2.1.1. Subjects

17 children with T1D were recruited for the study. Inclusion criteria were T1D diagnosed at least 6 months before the study was conducted, age 7 to 16 years, insulin treatment consisting of multiple daily injections (MDI) or continuous subcutaneous insulin infusion (CSII), and written informed consent by the participants and/or parents. The responsible Ethics Committee approved the study with ethics number 2020-00543.

#### 2.1.2. Experimental protocol

The recruited children participated in a prospective observational study in the setting of a one-week day camp (10 a.m., first study day to 10 a.m., seventh study day). In the camp, the children were supervised during the day by study physicians (from 9 a.m. to 5 p.m.). In the evenings and during the nights, the measurements and the logbook were continued at home. The first study day consisted of a trip to a climbing hall. On study days two to six, the children participated in various structured sports activities, divided into morning and afternoon sessions. The last study day consisted of collecting the devices.

In this camp setting, the activities, insulin treatment, and nutrition were controlled for all camp participants: Insulin doses (type, time, units), carbohydrate intake, type and duration of physical activity, and symptoms of hypoglycemia together with self-monitoring blood glucose (SMBG) (Section 2.1.3) were noted in a logbook by the study team.

#### 2.1.3. Hardware equipment and sensor setup

The hardware equipment for the data acquisition consisted of a glucose sensor (intermittently scanned continuous glucose monitoring (isCGM), Freestyle libre 2 (Abbott Diabetes Care Inc., Alameda, US) or a continuous glucose monitoring (CGM) device, Dexcom (Dexcom, San Diego, US)). The sensor was inserted into the subcutaneous tissue of the upper arm or on the abdomen. The glucose data were saved every 5 (CGM) or every 15 min (isCGM). Participants using the isCGM system were further able to conduct measurements when scanning the glucose device. The glucose measurements were completed with SMBG that were manually noted in a logbook. SMBG were conducted: each time symptoms of hypoglycemia were observed, when sensor measurements were below 3.9 mmol/l or above 15 mmol/l, before and after physical activity, and hourly during physical activity. Additionally, the logbook contains records about physical activity, insulin dosing, and meals. The exact features are described in Section 2.1.4.1. The glucose sensor was worn day and night.

#### 2.1.4. Preprocessing

Of the recruited children, one child dropped out of the study. The data of two children were not included in the analysis due to the usage of a hybrid closed-loop insulin pump, leaving 9 children with an isCGM device and 5 with a CGM device in the dataset.

To extract a single multivariate time series per child, the glucose sensor data (CGM or isCGM) needed to be merged with the SMBG, and the records about injected insulin doses, physical activity, and meals from the logbook. Short- and long-acting insulin doses were considered separately. Further, reported carbohydrates (CHO) were divided into three types: ones with fast absorption rate (e.g., glucose tablets or orange juice), slow absorption rate (e.g., whole grain or fatty products), and mixed absorption rate, i.e., full meals. Last, physical activity was reported in minutes. We merged both time series and overwrote CGM or isCGM measurements with SMBG values if they had identical time stamps. Further, we removed duplicate timestamps and kept the one with the lower glucose measurement.

After merging the time series data from both files, we curated all time series such that they have a sampling frequency of 5 min, where we use time-weighted linear interpolation to assign the glucose measurements to a time stamp, and summed the remaining measurements, i.e., if two meals were consumed during a 5 min period we assigned the sum of carbohydrates to the next time stamp.

We defined the start of a day as 7 a.m., which we later used to perform a sensible training/validation/testing split. Since glucose values were already available from 7 a.m. of the first study day, we included these values in the analysis and cut the data at 6:55 a.m. on the last study day to get a time series of 6 days. Note that this applies to all but one participant (13/14), for which the glucose measurements were only available for the last four days.

##### 2.1.4.1. Feature selection

Each model had access to all time-varying features obtained from the preprocessing described in Section 2.1.4, i.e., glucose levels in mmol/l, short-acting insulin, long-acting insulin in IE, physical activity in minutes, and carbohydrates of types in grams: with fast, slow and mixed absorption rate. Thus, the time series consists of 7 features.

Apart from time series records, we also used 11 participant characteristics: gender, age, weight, height, BMI, as well as diabetes specific features: time since the T1D diagnosis, percentage of basal insulin insulin dose, total daily basal, hemoglobin A1c (HbA1c), total daily dose of insulin (U/kg/d), and calculated total daily dose (computed as units per kilogram of weight). From all 11 features, the 6 features, i.e., gender, age, weight, BMI, the time since the T1D diagnosis, and the percentage of basal insulin, have been manually selected as a subgroup of features that we expected to be more relevant. In Section 3., we compare the recurrent architectures only using these 6 features to the ones that have access to the complete set of static features.

##### 2.1.4.2. Train test and validation split

We split the time series data for each participant individually into a train, validation and test set, and consider the static information to be present in each of these sets. We select the last 16.66% of time series as the test set, corresponding to a full day (288 time points) for each participant.^1^ Likewise, we cut off another 16.66% from the end of the time series for validation and leave the remainder of the dataset for training. Although this split leaves us with relatively few training days (4), we decided against using a smaller test set since otherwise, we cannot evaluate a full test day. Similarly, if we cut the validation set to a smaller size, it could, for example, only contain data from during the night, which would not be representative of the full time series.

### 2.2. Machine learning

In the following, we briefly describe the machine learning models that we evaluate on our data set, as well as explain how we extend them to also consider static data. After that, we describe the baselines, hyperparameter selection and evaluation metrics.

#### 2.2.1. Dilated RNN

**Figure 1:**
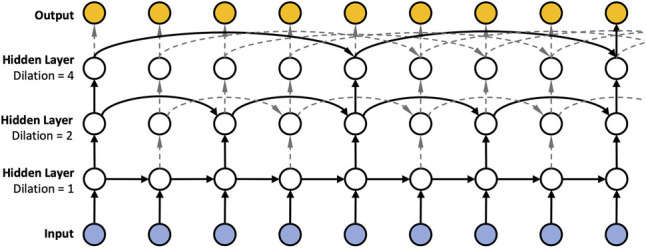
Three-layer dilated RNN with dilation 1, 2, and 4, and recurrent skip connections.

Since insulin dosing, as well as physical activity can have long ranging impact on blood glucose, Zhu et al. (21) proposed to use dilated RNNs (DRNNs) (35) in the context of blood glucose forecasting to account for such long ranging dependencies. DRNNs are akin to the dilated convolutions (20) employed in the field of computer vision, which seek to solve the vanishing gradient problem. Instead of using a “spatial” dilation as in dilated convolutions, DRNNs employ a “temporal” dilation (cf. Figure 1^2^). In particular, the previous state in a layer of a dilated RNN is not fetched from the previous time step but rather by skipping a predetermined number of time steps, or dilation rate. The dilation rate is set to 1 in the first layer and increases exponentially in subsequent layers, reaching 2 in the second layer, 4 in the third layer, and so on. Consequently, we can have different temporal resolutions in each layer and thus shorten the average recurrence path length between two samples due to the exponentially increasing dilation. In addition, DRNNs are fast to train (compared to dilated convolutions) since the sub-sequences generated by the dilation rate allow for parallelization.

We implemented dilation with RNN units, GRU (36) and LSTM units (37). We refer to the dilated versions as DRNN, DGRU, and DLSTM.

#### 2.2.2. (D)RNN with static features

As described in Section 2.1.4.1, we have access to a set of 11 static features characterizing each child in the cohort. Since it is known that, for example, insulin demands, as well as exercise and eating habits, depend on age and developmental stage (31), we expect that integrating these features is beneficial for forecasting. Building upon Miebs et al. (38), which investigated the integration of static features in RNNs, we investigate three distinct strategies for incorporating static characteristics into (D)RNNs: initialization of the hidden state, concatenation with the output, and the combination of both.

The first method utilizes the static features to initialize the hidden state of the DRNN. That is, let $h_{-1}$ denote the hidden state of the network at initialization, i.e., at time step t=0. We set $h_{-1}$ as

(1) $$h_{-1}={\text{MLP}}_{\text{init}}(s),$$

where s is our static feature vector associated with the current time series (e.g., the training time series of a certain child) and ${\text{MLP}}_{\text{init}}$ is a simple feed-forward neural network consisting of a single hidden layer with a ReLU activation function. We set the size of the hidden and the output layer equal to the size of the hidden state of the corresponding (D)RNN. Note that for all architectures involving LSTMs, we need to update both the hidden state $h_{-1}$ as well as the cell state $c_{-1}$ (39), which we do by using distinct MLPs of equivalent size.

As an alternative, we first transform the static features with a feed-forward network (${\text{MLP}}_{\text{cat}}$), then concatenate its output with the output $o_{t}$ of the RNN at time t and apply ${\text{MLP}}_{\text{head}}$ to the concatenated vector, i.e.,

(2) $${\hat{y}}_{t}={\text{MLP}}_{\text{head}}(o_{t},{\text{MLP}}_{\text{cat}}(s)),$$

where ${\text{MLP}}_{\text{cat}}$ consists of a single hidden state of size 2⋅#s, with #s denoting the number of static features, and output dimension two. ${\text{MLP}}_{\text{head}}$ consists of two hidden layers of size $2\cdot \#h_{t}$ and $\#h_{t}$. Both MLPs use ReLU activation functions.

**Figure 2:**
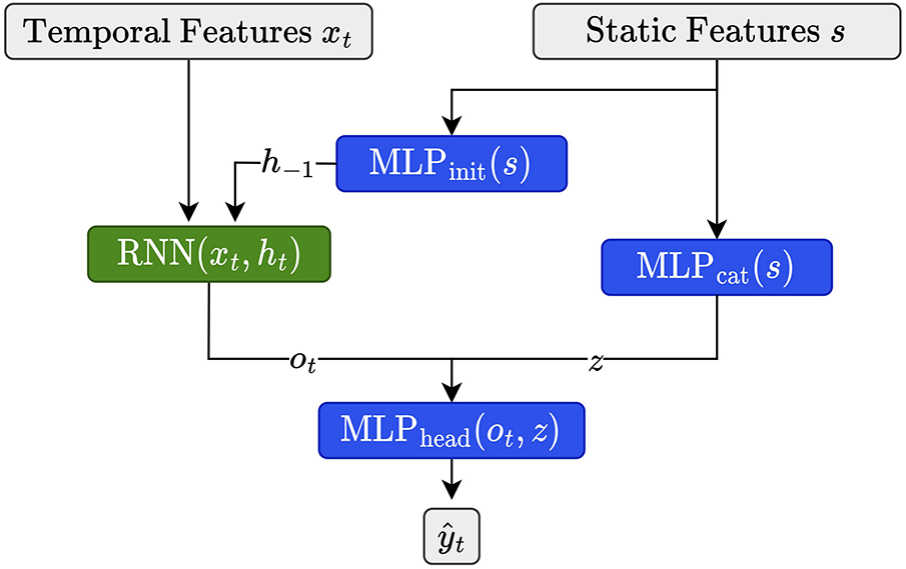
Illustration for static feature integration for RNN units using the combination of the initialization and output concatenation.

Finally, we simply combine both strategies as illustrated in Figure 2. We implemented all three strategies for dilated and non-dilated recurrent architectures.

#### 2.2.3. Temporal fusion transformer

Due to its strong empirical performance on several benchmark tasks, we additionally investigated the temporal fusion transformer (TFT) (40). Similar to our approach above, it can consider both temporal, as well as static features. Below, we provide a brief summary of the architecture and its training.

**Figure 3:**
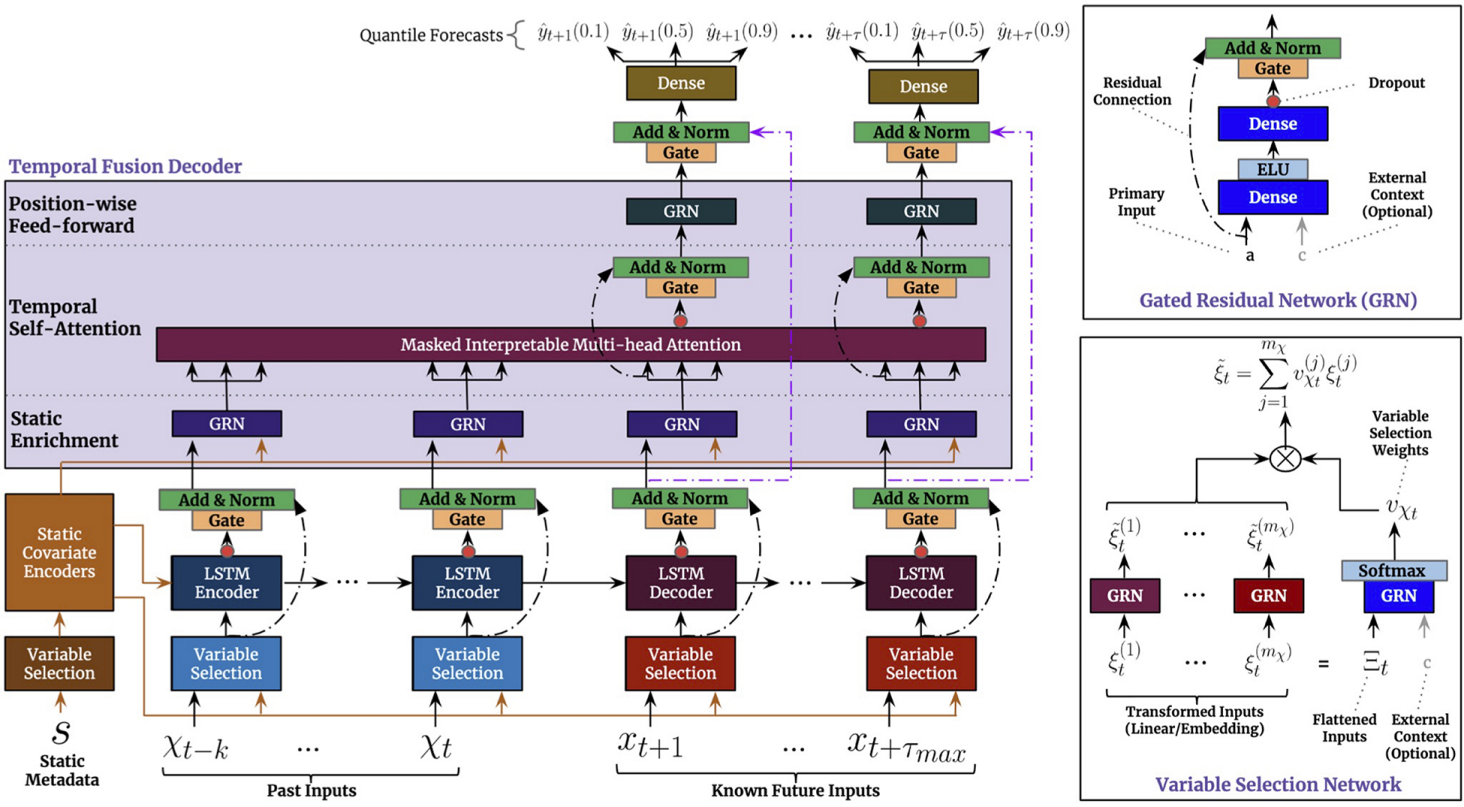
Illustration of the architecture of the Temporal Fusion Transformer.

We show the visualization of the architecture from the original paper (40) in Figure 3. In summary, the TFT is an encoder-decoder architecture that can consider three types of input: static inputs, past time-dependent inputs, and known future time-dependent inputs. In our case, the latter only consists of the time points that we want to predict.

To process the different types of input, the architecture uses a variable selection network (VSN) for each input type. The VSN is a neural network that selectively picks out the most important features for each time step, depending on the type of input. After the VSN processes the inputs, the past and future time-dependent inputs are fed into an LSTM encoder-decoder network. Finally, the processed inputs are sent through a multi-head attention network, which helps the model to attend to the most relevant information in the inputs. This allows the model to make predictions based on the most important information from the inputs. The static features processed by the affiliated VSN, are incorporated in the architecture at three distinct places: as supplementary input of the VSNs of the past inputs and the future known inputs, as initialization of the LSTM encoder-decoder structure, and as static enrichment for each timestamp inputted to the multi-head attention. Lastly, a gating mechanism is applied to skip over unused components of the architecture.

##### 2.2.3.1. TFT configuration

Besides providing a point prediction, TFT also generates prediction intervals (a set of quantiles) ${\hat{y}}_{q}$ with q∈Q={0.02,0.1,0.25,0.5,0.75,0.9,0.98}. To train the architecture we minimize the quantile loss, i.e.

(3) $$L(\Omega ,W)=\sum_{y_{t}\in \Omega }\sum_{q\in Q}\frac{QL(y_{t},{\hat{y}}_{q},q)}{N}$$

(4) $$QL(y,\hat{y},q)=q(y-\hat{y}{)}_{+}+(1-q)(\hat{y}-y{)}_{+}$$

where $(\cdot {)}_{+}$ refers to max(0,⋅), y is the ground truth value (e.g., the glucose value in 30 min), Ω is the domain of training data containing N samples, and W represents the weights of TFT.

To configure the architecture for our setting, we experiment with two different sizes of the look-back window (number of considered past inputs k in Figure 3), i.e. 2 and 12 h corresponding to 24 and 144 time steps, respectively. We set the decoder length to one, to only predict a single future value.

#### 2.2.4. Baselines

In the following paragraphs, we introduce the baselines and the range of hyperparameters that were used for the evaluation.

##### 2.2.4.1. Reference models

We consider two simple baselines that are common in the literature: *Identity* is implemented as the identity function, i.e., it predicts ${\hat{y}}_{t+1}$ as $y_{t}$. Additionally, we consider a *linear* model, which we implemented as a one-layer MLP without activation functions, where the input consists of all time-varying features.

##### 2.2.4.2. Recurrent neural networks

As the simplest deep learning architecture, we consider three types of recurrent neural networks (RNNs): vanilla RNN, gated recurrent units (GRUs) (36) and long short-term memory networks (LSTMs) (37).

#### 2.2.5. Hyperparameter selection

For all recurrent architectures, including the baselines, the dilated architectures, and recurrent architectures that incorporate static information, we set the learning rate to $10^{-3}$ and use a weight decay of $10^{-3}$. Due to preliminary testing, we observed that deeper RNN architectures did not improve the performances, which matches previous observations (21), hence, we restrict the hyperparameter search to hidden layers of size {3,5}, with each hidden layer consisting of {16,32} neurons. We use a batch size of 256 akin to previous work (21) since it enables us to utilize better the memory provided by the internal cell state of RNN architectures. All models are trained for 200 epochs, minimizing the mean squared error (MSE) for the training and validation set.

To evaluate the performance of the temporal fusion transformer, we set the learning rate to $10^{-3}$, use gradient clipping with a cutoff of 0.1, and select the number of hidden layers from {64,128}, the number of attention heads from {2,4}, the dropout from {0.2,0.4}, and the batch size from {32,256}. We train the transformer for at most 50 epochs, minimizing the recommended quantile loss with seven quantiles (40), and use early stopping with a cutoff of $10^{-4}$.

An overview of the hyperparameter grids and the size of the corresponding networks is provided in Table 1. Considering the recurrent architectures, the vanilla RNN has the fewest parameters, while the LSTM with static feature extension corresponds to the largest network of that type.

**Table 1:** Details with regard to model architecture, hyperparameter grid and train and inference time.

Model	Hyperparameter grid	#Parameters	Training time (s)	Inference time (s)
RNN/GRU/LSTM	#Layers ∈{3,5}	[1.5k,39k]	89.10±8.35	2.30±0.06
	#Neurons ∈{16,32}			
	Batch size: 256			
RNN+/GRU+/LSTM+	Epochs: 200	[1.9k,47k]	107.75±11.78	3.01±0.44
	Weight decay: $10^{-3}$			
	Learning rate: $10^{-3}$			
DRNN/DGRU/DLSTM	#Layers ∈{3,5}	[1.5k,39k]	148.26±36.77	6.67±1.62
	#Neurons ∈{16,32}			
	Batch size: 256			
DRNN+/DGRU+/DLSTM+	Epochs: 200	[1.9k,47k]	170.66±38.75	7.48±1.73
	Weight decay: $10^{-3}$			
	Learning rate: $10^{-3}$			
TFT	#Neurons ∈{64,128}	[453k,1775k]	653.10±146.92	29.86±3.02
	#Attention heads ∈{2,4}			
	Dropout (%) ∈{20,40}			
	Batch size ∈{32,256}			
TFT+	Gradient Clipping: 0.1	[635k,2465k]	741.11±153.61	35.11±3.07
	Epochs: 50			
	Learning rate: $10^{-3}$			

The number of parameters is reported as [min,max] with respect to the hyperparameter grid. Training and inference time are reported as mean and standard deviation over all models contained in the grid, where we considered the full day prediction task with a prediction horizon of 30 min (cf. Section 3.3). Models with a “+” correspond to extended models with static feature integration.

#### 2.2.6. Model training

To train each model, we perform a grid search for which we specify the hyperparameter grid in Section 2.2.5. As standard, we use the training split to update the parameters of the model and the validation split to select the best hyperparameters. Since we have access to at most four days of records per participant, we decided to train a population model.

The training is composed of two nested loops: the first of which iterates over the epochs while the second iterates through all participants, where we shuffle the participant order at the start of each epoch. For any RNN architecture, we reset the hidden state before iterating through the training data of a new participant. The validation loss is aggregated over all participants. When training the transformer, we instantiate the corresponding training scheme via the “PyTorch Forecasting” library (41). Further, we employ early stopping for TFT with patients of 5 epochs and a minimum delta of $10^{-4}$ based on the validation performance.

All models are implemented in PyTorch and trained with the Adam optimizer (42). For the deep-learning architectures, all features are standardized based on the training set, and we select the best hyperparameters based on the validation loss. The training was conducted on GPU (NVIDIA GeForce RTX 2080 Ti). We report training and inference times for the main results with a prediction horizon of 30 min in Table 1. The reported times correspond to an average model within the grid. Training times for the transformer have a large variance due to the early stopping. The longest training time for a single parameter configuration of a transformer model was approximately 21 min.

#### 2.2.7. Evaluation metrics

To evaluate the performance of the considered models, we use three common metrics: the root mean squared error (RMSE), the mean absolute prediction error (MAPE), and the $R^{2}$ metric, defined as

(5) $$\text{RMSE}(\hat{y},y)=\sqrt{\frac{1}{T}\sum_{t=1}^{T}(y_{t}-{\hat{y}}_{t}{)}^{2}}$$

(6) $$\text{MAPE}(\hat{y},y)=\frac{1}{T}\sum_{t=1}^{T}\left(\frac{\left|y_{t}-{\hat{y}}_{t}\right|}{y_{t}}\right)$$

(7) $$R^{2}(\hat{y},y)=1-\frac{\sum_{t=1}^{T}(y_{t}-{\hat{y}}_{t}{)}^{2}}{\sum_{t=1}^{T}(y_{t}-\bar{y}{)}^{2}}.$$

with $\bar{y}$ being the mean value of y. Further, we consider Parkes error grids (43), which is a standard in Glucose forecasting, to visualize the results of the best performing methods. We provide further details for the interpretation of Parkes error grids in the corresponding section.

## 3. Results

Next, we present the results on our dataset. First, we show the patient characteristics of the cohort included after preprocessing. Then we analyze the effect of the static feature integration in Section 3.2, and show a comparison of the best RNN, dilated RNN architectures, as well as for TFT for different forecasting horizons in Section 3.3. We further visualize the effect of the static data integration for the RNN architecture via the common Parkes error grids in Section 3.5.

### 3.1. Participant characteristics

A summary of the participant characteristics (after preprocessing) is provided in Table 2. The data included 14 children with T1D aged 7–13 years old, from which 5 (∼36%) were female. All features show a relatively high variance, motivating the use of static features for a population model or in cases where more data is available, a personalized approach. The BMI, which is related to height and weight, ranges from 13.2 to 27.7 kg/m${}^{2}$. The average time since T1D diagnosis was 3.8 years, ranging from half a year to nine 9 and a half years. Glycated hemoglobin (HbA1c) was measured in the range of 79 days before the first study day and 74 days after the first study day, and ranges from 5.1 to 8.5. Further, the basal insulin percentage lies between 26.2 and 69.4% and the total daily calculated insulin between 0.31 and 1.65 (U/kg/d).

**Table 2:** Participant characteristics for all N=14 participants (5/14 female).

	Mean ± Std	Range
Age (years)	11.2±2.1	[7.5,13.9]
BMI (kg/m${}^{2}$)	19.5±4.2	[13.2,27.7]
Weight (kg)	45.1±16.5	[21.0,77.8]
Height (cm)	149.4±14.1	125.3,171.0
Duration of diabetes (years)	3.8±2.8	[0.5,9.5]
HbA1c (%)	7.2±0.8	[5.1,8.5]
Basal insulin (%)	46.1±12.5	[26.2,69.4]
Total daily calculated insulin (U/kg/d)	0.90±0.39	[0.31,1.65]

### 3.2. Effect of static feature integration

**Figure 4:**
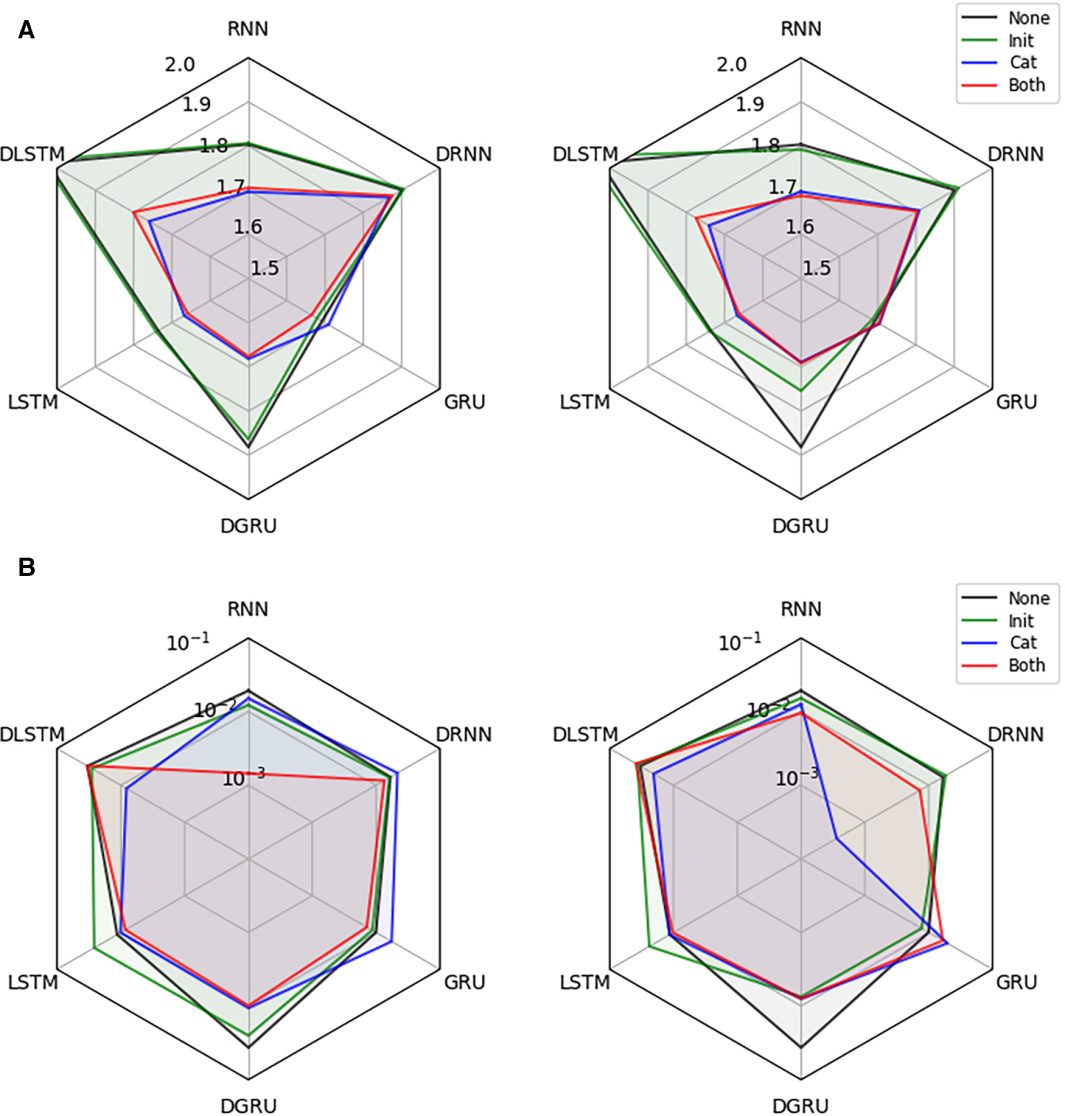
Effectiveness of static data integration: Subfigure (**A**) shows the test RMSE over 3 seeds for the best performing RNN, DRNN, GRU, DGRU, LSTM, and DLSTM (selected on validation performance) considering no static feature integration (None), concatenation of outputs (Cat), initialization of the hidden state (Init) and the combination of both techniques (Both). The left plot uses the hand-selected 6 static features and the right plot all 11 static features. Subplot (**B**) shows the corresponding standard error.

Next, we investigate the effectiveness of integrating static information into the recurrent architectures. We conduct the experiment on the dataset described in Section 2.1, for a prediction horizon of 30 min and allow for a lookup window of two hours (24 time points), i.e., the first 24 test points for each participant are not considered for computing the loss but only to update the hidden state of the models. For each combination of network architecture and type of static data integration, we show the test RMSE in mmol/l (mean over 3 seeds) and the corresponding standard error of the best performing model, which was selected based on the validation performance, in Figure 4. All results are shown for models with access to all static features, and when they only have access to the six hand-selected features.

We see that static feature integration clearly helps improve most models with DRNN and vanilla GRU being the exceptions. From the different variants of feature integration, *Cat* (concatenation strategy) and *Both* (initialization and concatenation) outperform the *Init* (initialization strategy) strategy. Since all methods perform almost identically for *Cat* and *Both*, as well as for *Init* and no static feature integration, we conclude that concatenation of static features is responsible for the majority of the performance improvement. The overall best performing methods are the LSTM and GRU with concatenation, both reaching an RMSE of 1.66 mmol/l. The vanilla RNN, as well as the dilated GRU with concatenation reach an RMSE of 1.71 and 1.68 mmol/l, respectively. Especially the RNN seems to benefit from the static feature integration, improving the RMSE by 0.09 mmol/l, while the improvements for the LSTM and GRU are 0.08 and 0.03 mmol/l, respectively. Although all dilated architectures seem to significantly improve with static feature integration, they do not outperform the vanilla architectures. We suspect that this is due to the limited length of the time series that are available during training, which may lead to an underfitting of these architectures.

### 3.3. Evaluation for increasing prediction horizon

Next, we evaluate the performance of the best-performing RNN architectures from the evaluation in Section 3.2, i.e., RNN, LSTM, GRU and DGRU, against the baselines and the temporal fusion transformer (TFT). Further, we consider the corresponding extended networks that use the combined strategy (*Both*) for static feature integration, denoted as RNN+, LSTM+, GRU+, and DGRU+. Similarly, we add a version of TFT, TFT+, which has access to all static features. We consider two different settings: one which considers the full test day, for which we allow a lookup window of 2 h (the first 24 data points do not affect the test performance), and a second one in which we set the lookup window to 12 h (144 data points). The latter corresponds to a window in the evening and night (7 p.m. to 6:55 a.m. at the next day), which is of high interest as participants are not actively monitoring their glucose levels during the night.

We show mean and standard deviation over 3 seeds in Table 3. As before, hyperparameter selection was done based on the validation performance. Although LSTM+ and GRU+ have the best performance for a forecasting window of 30 min in both tasks, RNN+ has the best overall performance, i.e. performing best for a forecasting window of 60 and 120 min. Further, RNN+ clearly outperforms the RNN version without static feature integration for every setting. In general, all methods with static feature integration match or outperform their counterparts for a forecasting window of 30 min, while this is not always the case for the LSTM, GRU and TFT for PH 60 and 120. Despite not having the best RMSE in any setting, TFT and TFT+ perform well in terms of MAPE, where TFT+ performs best for PH 60 and is within two standard errors of the best method for PH 30 for a lookup window of two hours. The performance for a prediction horizon of 120 min is poor for all methods, whereas RNN+ is the only method that consistently outperforms the linear baseline in this setting.

**Table 3:** We report the mean and standard error of the test RMSE in mmol/l, MAPE and $R^{2}$ over three seeds for increasing prediction horizons (PH) from 30 to 120 min. Table [Full Day] shows the results on the full test set with a lookup window of 2 h. For table [Night] the lookup window is equal to 12 h, i.e., predictions start after 7 pm. We mark the best result in bold and underline statistically indistinguishable results. If a method does not perform better than the linear baseline, we highlight it in gray.

Full Day			
Model	RMSE	MAPE	$R^{2}$
PH=30 min			
Identity	2.03	20.10	0.60
Linear	1.94±0.00	19.95±0.04	0.64±0.00
TFT	1.75±0.01	17.02±0.21	0.71±0.01
TFT+	1.74±0.01	$\underline{16.88\pm 0.21}$	0.71±0.00
RNN	1.80±0.02	18.48±0.22	0.69±0.01
RNN+	1.71±0.00	17.06±0.08	0.72±0.00
LSTM	1.74±0.01	17.63±0.25	0.71±0.00
LSTM+	$\underline{1.66\pm 0.01}$	$\underline{16.76\pm 0.09}$	$\underline{0.74\pm 0.00}$
GRU	1.69±0.01	17.11±0.11	0.72±0.00
GRU+	$\underline{1.66\pm 0.01}$	$\underline{16.94\pm 0.12}$	0.73±0.00
DGRU	1.88±0.04	19.43±0.68	0.66±0.01
DGRU+	$\underline{1.68\pm 0.01}$	17.12±0.19	0.73±0.00
PH=60 min			
Identity	2.83	27.95	0.19
Linear	2.59±0.00	26.76±0.07	0.32±0.00
TFT	2.49±0.01	$\underline{24.88\pm 0.13}$	0.37±0.00
TFT+	2.45±0.01	$\underline{24.79\pm 0.38}$	0.39±0.01
RNN	2.67±0.02	28.44±0.59	0.27±0.01
RNN+	$\underline{2.41\pm 0.01}$	$\underline{25.08\pm 0.65}$	$\underline{0.41\pm 0.00}$
LSTM	2.46±0.01	$\underline{25.43\pm 0.06}$	0.39±0.01
LSTM+	2.54±0.02	25.91±0.21	0.34±0.01
GRU	2.54±0.02	27.78±0.40	0.34±0.01
GRU+	2.56±0.06	27.29±0.99	0.33±0.03
DGRU	2.90±0.03	30.24±0.29	0.14±0.02
DGRU+	2.69±0.04	27.01±0.78	0.27±0.02
PH=120 min			
Identity	3.84	39.47	−0.56
Linear	3.31±0.01	35.32±0.09	−0.16±0.01
TFT	3.23±0.03	33.62±0.35	−0.10±0.02
TFT+	3.28±0.03	34.27±0.19	−0.14±0.02
RNN	3.15±0.07	35.66±1.45	−0.05±0.05
RNN+	$\underline{3.04\pm 0.03}$	$\underline{32.90\pm 0.16}$	$\underline{0.02\pm 0.02}$
LSTM	3.71±0.06	38.33±0.97	−0.46±0.05
LSTM+	3.54±0.11	37.01±0.83	−0.33±0.08
GRU	3.66±0.20	39.09±1.27	−0.43±0.16
GRU+	3.47±0.03	36.07±0.89	−0.27±0.02
DGRU	4.16±0.14	43.18±1.64	−0.83±0.12
DGRU+	3.74±0.17	38.20±1.28	−0.49±0.13

Night			
Model	RMSE	MAPE	$R^{2}$
PH=30 min			
Identity	1.84	16.28	0.64
Linear	1.77±0.00	16.09±0.04	0.67±0.00
TFT	1.56±0.01	13.69±0.12	0.75±0.00
TFT+	1.57±0.01	13.71±0.06	0.74±0.00
RNN	1.64±0.01	14.44±0.08	0.72±0.00
RNN+	1.55±0.01	13.89±0.05	0.75±0.00
LSTM	1.59±0.02	14.04±0.34	0.73±0.01
LSTM+	$\underline{1.50\pm 0.01}$	$\underline{13.17\pm 0.13}$	$\underline{0.77\pm 0.00}$
GRU	1.57±0.01	13.77±0.17	0.74±0.00
GRU+	$\underline{1.50\pm 0.02}$	$\underline{13.34\pm 0.18}$	0.76±0.01
DGRU	1.76±0.03	15.86±0.48	0.67±0.01
DGRU+	$\underline{1.51\pm 0.01}$	13.46±0.11	0.76±0.00
PH=60 min			
Identity	2.48	21.53	0.33
Linear	2.30±0.00	20.58±0.07	0.43±0.00
TFT	2.22±0.04	19.59±0.31	0.46±0.02
TFT+	2.28±0.06	19.64±0.40	0.43±0.03
RNN	2.34±0.07	21.64±0.87	0.41±0.04
RNN+	$\underline{2.10\pm 0.05}$	$\underline{18.93\pm 0.34}$	$\underline{0.52\pm 0.02}$
LSTM	$\underline{2.15\pm 0.01}$	$\underline{18.86\pm 0.17}$	$\underline{0.50\pm 0.01}$
LSTM+	$\underline{2.18\pm 0.03}$	19.22±0.34	$\underline{0.48\pm 0.01}$
GRU	2.23±0.01	21.03±0.20	0.46±0.01
GRU+	2.32±0.08	21.11±1.17	0.41±0.04
DGRU	2.61±0.11	23.82±0.75	0.26±0.06
DGRU+	2.28±0.02	20.21±0.70	0.44±0.01
PH=120 min			
Identity	3.36	30.11	−0.41
Linear	2.89±0.01	$\underline{26.57\pm 0.07}$	$\underline{-0.04\pm 0.01}$
TFT	3.13±0.02	28.94±0.20	−0.22±0.01
TFT+	3.02±0.10	27.14±0.92	−0.15±0.07
RNN	$\underline{2.79\pm 0.09}$	$\underline{26.61\pm 1.39}$	$\underline{0.02\pm 0.06}$
RNN+	$\underline{2.70\pm 0.09}$	$\underline{24.89\pm 0.87}$	$\underline{0.08\pm 0.06}$
LSTM	3.07±0.05	28.92±0.60	−0.18±0.04
LSTM+	3.01±0.08	27.76±1.01	−0.14±0.06
GRU	3.25±0.04	30.79±0.08	−0.33±0.03
GRU+	2.95±0.20	27.31±2.09	−0.11±0.15
DGRU	3.58±0.19	34.59±2.20	−0.61±0.18
DGRU+	3.25±0.14	29.92±0.60	−0.33±0.12

When we compare the results on our dataset to the literature, we note that we do not reach the performance of comparable models on the OhioT1DM dataset (4) consisting of much longer time series per participant (8 weeks) and comprising only adults. In a recent benchmark (9), including deep learning and non-deep learning architectures, an LSTM was among the best-performing methods, reaching an RMSE of around 1.12 for a prediction horizon (PH) of 30 min and an RMSE of 2.64 for PH equal to 120 min.^3^ Further, the reference measure for the OhioT1DM dataset was reported to be 1.57 and 3.20 for PH equal to 30 and 120, which is much lower than for our dataset—indicating that the prediction task is harder for our dataset. In terms $R^{2}$, we observe encouraging results for PH 30. As a reference, a personalized LSTM model trained on in silico adult patients simulated with the UVA/Padova simulator (44), achieved an $R^{2}$ of 0.75 for a prediction horizon of 40 min (45). In comparison, we achieve an $R^{2}$ of 0.71 for the vanilla LSTM and 0.74 for the extended LSTM for PH 30 on our dataset trained on population data. For a prediction horizon of 120 min, however, can see that most models struggle to obtain any meaningful information. This negative result has also been observed for the OhioT1DM dataset (24) where $R^{2}$ values are equal to zero for some models, reaching at most 0.16 for the best-performing model.

When considering the prediction results during the night (Table 3 right), we see the same trend for the comparison between methods. At the same time, the RMSE improves for all methods, with the best performance for PH 30 improving to an RMSE of 1.50 mmol/l for LSTM+ and GRU+. For a prediction horizon of 60 and 120, the best performing method, RNN+, has an improved RMSE by 0.31 and 0.34 mmol/l, respectively. In terms of MAPE, the performance of RNN+ for PH 120 is on par with its performance for the full day with PH 60. To our surprise, the transformer did not improve as much as the RNN+ despite having access to a larger lookup window of 12h—which would allow it to capture longer ranging dependencies.

### 3.4. Participant-level results

Besides looking at the aggregated results, we also inspected the individual results per participant (in terms of test RMSE), which we show in Table 4. In particular, we show the results for PH 30 on the full day prediction task for all methods considered in Section 3.3. In contrast to the previous section, we first compute the mean and standard error over three seeds *per participant* and then compute an aggregated score shown in column *Average*, which is computed as the mean and standard deviation of the mean results among all participants.

**Table 4:** Shown is the mean and standard error of the test RMSE in mmol/l over three seeds for a prediction horizon of 30 for each participant in the study. Further, *Average* is the mean and *standard deviation* in brackets over the mean results of the participants.

Full Day															
Model	Average	ID-004	ID-005	ID-006	ID-007	ID-008	ID-009	ID-010	ID-011	ID-013	ID-014	ID-015	ID-016	ID-017	ID-020
Identity	1.90 (0.65)	1.96±0.0	1.44±0.0	1.88±0.0	1.50±0.0	3.75±0.0	1.61±0.0	2.07±0.0	2.17±0.0	2.62±0.0	1.98±0.0	1.42±0.0	1.13±0.0	1.11±0.0	1.98±0.0
Linear	1.82 (0.62)	1.91±0.0	1.35±0.0	1.76±0.0	1.47±0.0	3.58±0.0	1.56±0.0	1.97±0.0	2.05±0.0	2.50±0.0	1.88±0.0	1.37±0.0	1.09±0.0	1.09±0.0	1.90±0.0
TFT	1.64 (0.54)	1.12±0.0	1.00±0.0	1.98±0.1	1.34±0.0	2.69±0.0	2.70±0.1	1.04±0.0	1.20±0.0	1.98±0.0	1.23±0.0	1.53±0.0	1.73±0.0	1.48±0.0	1.88±0.0
TFT+	1.64 (0.54)	1.12±0.0	1.00±0.0	1.98±0.1	1.34±0.0	2.69±0.0	2.70±0.1	1.04±0.0	1.20±0.0	1.98±0.0	1.23±0.0	1.53±0.0	1.73±0.0	1.48±0.0	1.88±0.0
RNN	1.70 (0.54)	2.03±0.0	1.50±0.0	1.67±0.0	1.50±0.1	3.19±0.0	1.19±0.0	1.85±0.1	1.98±0.0	2.19±0.0	1.86±0.0	1.22±0.0	1.24±0.1	0.96±0.0	1.44±0.0
RNN+	1.61 (0.50)	1.96±0.1	1.25±0.0	1.63±0.0	1.29±0.0	2.94±0.1	1.14±0.0	1.72±0.0	1.80±0.0	2.14±0.1	1.83±0.0	1.24±0.0	1.26±0.0	0.91±0.0	1.42±0.1
LSTM	1.64 (0.52)	2.05±0.1	1.22±0.0	1.49±0.0	1.42±0.0	3.03±0.1	1.22±0.0	1.86±0.1	1.98±0.0	2.16±0.0	1.71±0.0	1.17±0.0	1.06±0.1	1.16±0.0	1.45±0.0
LSTM+	1.58 (0.45)	1.95±0.0	1.23±0.0	1.42±0.0	1.34±0.0	2.70±0.0	1.24±0.0	1.67±0.0	1.87±0.0	2.16±0.0	1.74±0.0	1.17±0.0	1.06±0.0	1.07±0.0	1.52±0.0
GRU	1.60 (0.50)	1.99±0.0	1.16±0.0	1.50±0.0	1.36±0.0	2.93±0.0	1.21±0.0	1.73±0.0	1.89±0.0	2.13±0.0	1.69±0.0	1.17±0.0	1.15±0.0	1.00±0.0	1.48±0.0
GRU+	1.59 (0.45)	1.96±0.1	1.28±0.0	1.46±0.0	1.33±0.0	2.71±0.0	1.26±0.0	1.69±0.0	1.92±0.0	2.12±0.0	1.73±0.0	1.17±0.0	1.05±0.0	1.04±0.0	1.49±0.0
DGRU	1.76 (0.62)	2.04±0.0	1.38±0.0	1.52±0.0	1.67±0.2	3.52±0.0	1.26±0.0	1.91±0.0	2.11±0.1	2.39±0.0	1.79±0.0	1.30±0.0	1.10±0.0	1.05±0.0	1.52±0.0
DGRU+	1.60 (0.46)	1.98±0.0	1.28±0.0	1.46±0.0	1.34±0.0	2.74±0.0	1.26±0.0	1.73±0.0	1.89±0.0	2.15±0.0	1.72±0.0	1.17±0.0	1.08±0.0	1.01±0.0	1.51±0.0

We see that the average performance among participants is slightly better compared to the results shown in Table 3, with the best performing method LSTM+ achieving an average RMSE of 1.58 mmol/l compared to 1.66 mmol/l shown in Table 3. Encouraging to see is that by using static feature extension, not only the average performance is improved (for the RNN, LSTM, GRU, and DGRU), but also the standard deviation is lower, which is exactly what we aimed to achieve. For example, for the LSTM, the standard deviation reduces from 0.52 to 0.45, with the mean performance improving from 1.64 to 1.58 mmol/l. On the other hand, we see that two participants (ID-008 and ID-013) have a particularly high error for the baseline measures *Identity* and *Linear*. A possible explanation for these results is that for participant ID-008, the study physicians needed to intervene more frequently to correct hypoglycemia than for other study participants, and participant ID-013 had particularly high blood sugar values that partially exceeded the limits of the measurement device (>33.3 mmol/l). Lastly, we can see a different trend between the transformer and the RNN architectures. That is, TFT and TFT+ perform slightly better on participants ID-008 and ID-013 while trading off this performance gain for other participants. This observation is in line with the lower MAPE values of TFT and TFT+ compared to the achieved RMSE that we saw in Table 3. We conjecture that this observation is due to the different loss, i.e., the quantile loss, that is used during the training of TFT.

### 3.5. Parkes error grids

**Figure 5:**
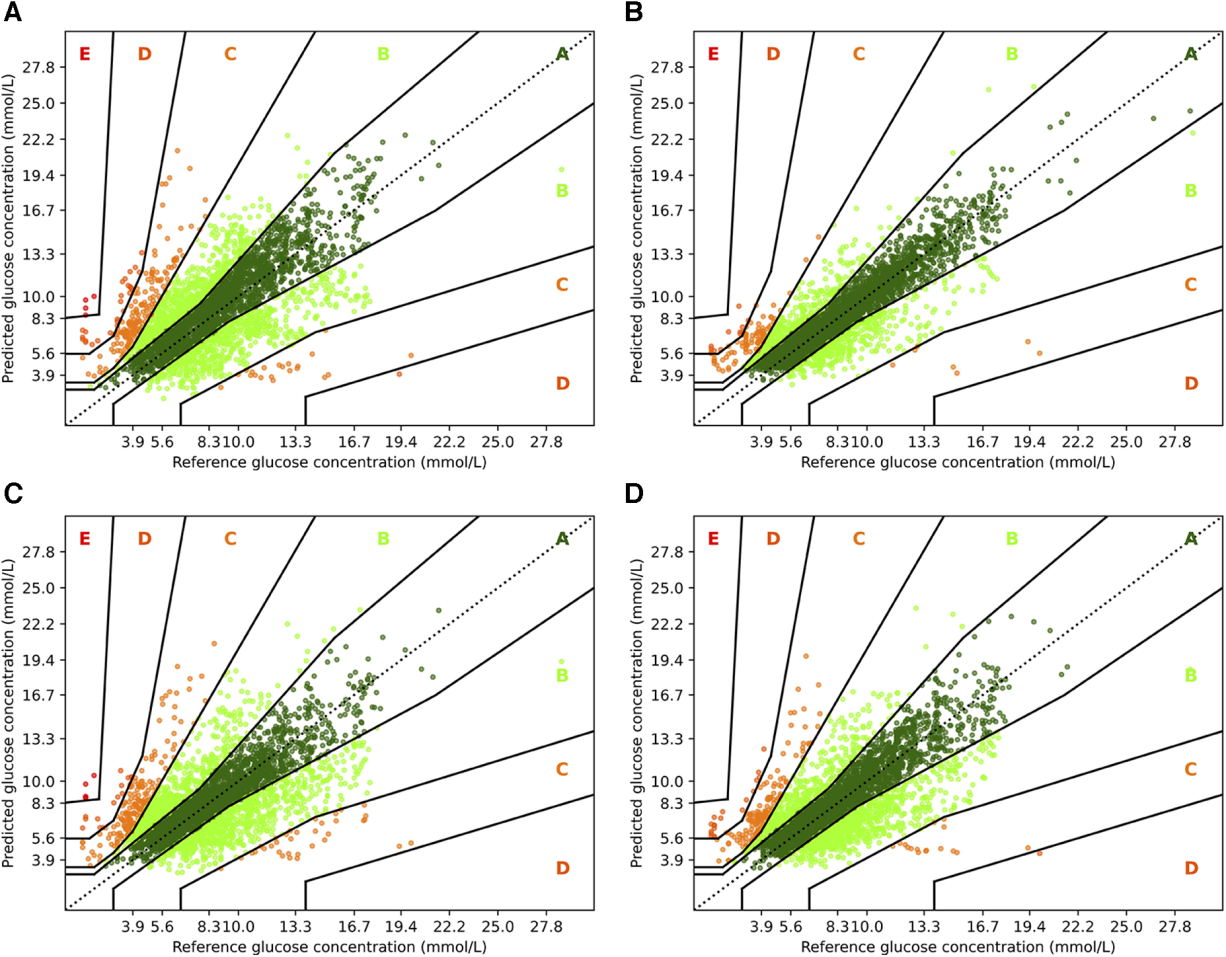
Parkes error grids for RNN with a prediction horizon of 30 min (**A**) resp. 60 min (**C**), and for RNN+ for PH 30 (**B**) and PH 60 (**D**). The a lookup window is 2 h.

**Figure 6:**
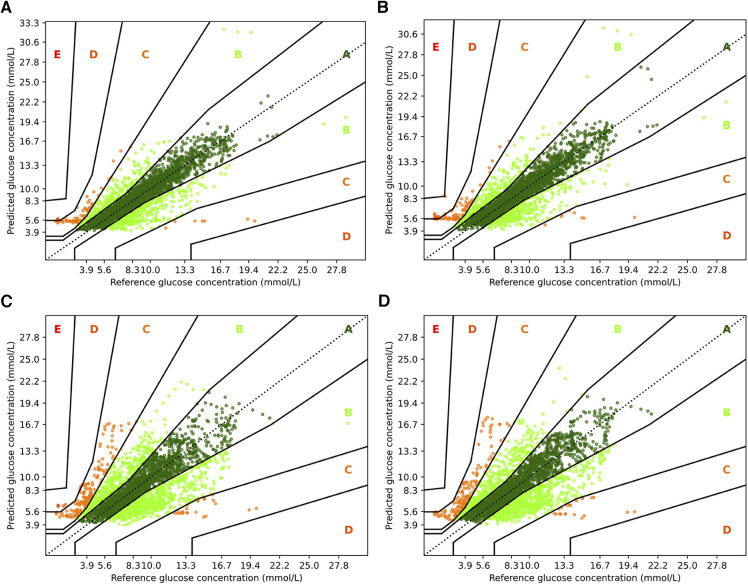
Parkes error grids for TFT with a prediction horizon of 30 min (**A**) resp. 60 min (**C**), and for TFT+ for PH 30 (**B**) and PH 60 (**D**). The a lookup window is 2 h.

To complement the analysis from the previous sections, we also show the Parkes error grids (43), which are a standard evaluation measure for methods focusing on glucose forecasting. It consists of a scatter plot of ground truth and predicted values overlaid with a grid of five zones with medical interpretation: Zone A represents no effect on clinical action; zone B represents altered clinical action—little or no effect on clinical outcome; zone C represents altered clinical action—likely to affect clinical outcome; zone D represents altered clinical action—could have significant medical risk; and zone E represents altered clinical action—could have dangerous consequences. We show the Parkes error grids^4^ for RNN and RNN+ for a prediction horizon of 30 and 60 min in Figure 5, and additionally show the plots for TFT and TFT+ in Figure 6.

First, we consider the results for RNN and RNN+. Overall, it is encouraging that the largest part of the predictions are in zones A and B. For PH 30, 77.72% of all predictions are within zone A, while no predictions are in zone E, and only 2.58% and 0.36% are in zones C and D. In contrast, a higher fraction (5.38%) of predictions for the vanilla RNN are in zone C, and 0.83% and 0.11% are in zones D and E, respectively. Also, the amount of predictions in zone A reduces significantly to only 55.86%. If we consider the forecasting window of 60 min, we observe a similar behavior. Naturally, both models have a lower fraction of predictions in zone A: 62.04% for RNN+ and 56.19% for the RNN. Further, 5.18% and 6.35% of predictions are outside of zones A and B with 4.63% and 5.82% percent in zone C.

For TFT and TFT+, we observe a similar trend between the two prediction horizons, but a smaller difference between the variants. When predicting 30 min ahead, both variants perform nearly identically assigning 77.41% and 77.25% to zone A, 19.92% and 20.06% to zone B and almost no predictions to zone D (0.08% and 0.33%, respectively). A slight advantage to the RNN architectures is that for PH 60, TFT+ has all predictions in zones A, B and C (approx. 60.97%, 33.71%, and 5.31%)—which is also the setting for which TFT+ has the best MAPE. TFT, however, assigns 0.28% to zone D, 5.45% to zone C, and 35.61% to zone B.

## 4. Discussion and conclusion

This study describes the approach to forecasting blood glucose levels in children with T1D using a dataset collected in a controlled day camp setting, where children participated in various physical activities. We investigated a) whether deep-learning models can achieve comparable results as reported on adult data, b) if integrating static patient information is beneficial for forecasting algorithms, and c) if the performance for full day prediction differs to the one obtained when only predicting during the night.

In a small dataset of children of various ages engaging in multiple types of physical activity, we were able to make a short term glucose prediction with acceptable accuracy—even during the night. This is of special relevance, as the risk for nocturnal hypoglycemia increases with time spent in physical activity, and the fear of hypoglycemia could be a barrier and prevent patients from exercising. Despite the improvements due to static feature integration, we could, however, not reach the performance of the LSTM and DRNN on the OhioT1DM dataset (5, 9, 21), where the discrepancies are most evident for a prediction horizon of 30 min. We find that especially the DRNN architecture does not perfrom well on our dataset. While an RMSE of 1.05 mmol/l was reported on the OhioT1DM dataset (35), the dilated approaches cannot outperform their non-dilated counterparts in our evaluation. When comparing our dataset to the OhioT1DM dataset, we identified several aspects that can contribute to this discrepancy. First, the time series in our dataset are significantly smaller (i.e., 6 days compared to 8 weeks), which mostly affects the deep learning architectures, whereas the effect on the baselines is minor. Second, the children in our study conducted several hours of exercise per day, which is known to have both short and long-lasting effects on the blood glucose levels (32, 34), thus inducing a higher complexity in the dynamics influencing the blood glucose level. An additional factor could be the heterogeneity of the study group (cf. Section 3.1), as age and developmental stage of a child influence insulin sensitivity and insulin requirements (31). In line with these characteristics of our dataset, we also report larger values for RMSE and MAPE for the reference measure compared to values reported for the OhioT1DM dataset. For example, for a prediction horizon of 30 min, we report an RMSE of 2.03 mmol/l, whereas an RMSE of 1.57 mmol/l has been reported for the OhioT1DM dataset. Similarly, the corresponding values for MAPE are 20.10% and 13.51%, respectively.

Regarding the integration of static features, our results clearly show that this adjustment leads to an improved test performance, especially for the vanilla RNN. Among the three strategies of static feature integration that we implemented, a simple output concatenation proved to be the most effective strategy. The overall best performing method was the RNN+, which integrated the static features via concatenation. For short term forecasting (30 min) the LSTM and GRU networks with static feature integration had a slight advantage. For the transformer architecture, the static feature integration did not lead to substantial improvements, however, it stabilized the approach in terms of MAPE with PH equal to 30. If we consider the dilated approaches, we see that static data integration leads to strong improvements in some cases. Since these methods generally had a compromised performance on our dataset, however, one needs to be careful with drawing conclusions from those results. We think that both the transformer, as well as the DRNN would benefit from larger time series in the training set, as was demonstrated for DRNNs on the OhioT1DM dataset (21). In addition, the signal about physical activity might be too sparse for the models to pick it up for estimating long ranging dependencies. An interesting avenue for future work would be to investigate if pre-training those approaches on larger datasets improves their performance. A major hurdle for this experiment is, however, that there exists no larger dataset which matches our feature set.

Lastly, if we compare the performances for the full day to the night, one would expect the results during the night to be better since it is well known that closed loop insulin pumps work best during the night. Moreover, there are fewer factors influencing blood glucose during this period, i.e. no meals are consumed and no physical activity is conducted. Our results confirm this expectation for prediction horizons of 30 and 60 min. For predicting two hours ahead, however, none of the method delivers convincing results. While the results for short-term prediction are encouraging, the long term prediction shows that we are far from being able to make a reliable prediction in the evening for whether blood glucose levels will reach a critical level during the night.

We aim for our study to inspire further research dedicated to enhancing diabetes care for children with T1D in the future. From our study, we see that blood glucose forecasting in children conducting regular physical activity is challenging, especially if one aims to predict multiple hours ahead. Besides collecting larger datasets with potentially longer time series, we think that an interesting aspect for future work lies in a more fine grained modelling of physical activity. For instance, one could integrate information from a wearable, such as heart rate (cf. (46, 47)), to estimate the intensity of physical activity to provide the models with more information about the status of a participant.

## Data Availability

The data analyzed in this study is subject to the following licenses/restrictions: The datasets presented in this article are not readily available to ensure participant privacy. Requests to access these datasets should be directed to MAB or SB.

## Footnotes

^1^These numbers count for all participants for which we have data for the full study period of 6 days, i.e., all but one for which only data for 4 days was available.

^2^Figure taken from the original paper (35).

^3^For the OhioT1DM dataset the participants are evaluated individually and the RMSE is the average among participants. In our setting, we do not average per participant, but simply by the number of test points or all participants.

^4^The plots were created with the Python library *methcomp* version 1.0.1.
